# Collecting Multi-country Retrospective Antimicrobial Consumption and Use Data: Challenges and Experience

**DOI:** 10.1093/cid/ciad667

**Published:** 2023-12-20

**Authors:** Kristi Prifti, Kyu-young Kevin Chi, Emmanuel Eraly, Hea Sun Joh, Mohammad Julhas Sujan, Nimesh Poudyal, Florian Marks, Marianne Holm

**Affiliations:** International Vaccine Institute, Seoul, Republic of Korea; International Vaccine Institute, Seoul, Republic of Korea; International Vaccine Institute, Seoul, Republic of Korea; International Vaccine Institute, Seoul, Republic of Korea; International Vaccine Institute, Seoul, Republic of Korea; International Vaccine Institute, Seoul, Republic of Korea; International Vaccine Institute, Seoul, Republic of Korea; Cambridge Institute of Therapeutic Immunology and Infectious Disease, University of Cambridge School of Clinical Medicine, Cambridge, United Kingdom; Heidelberg Institute of Global Health, University of Heidelberg, Heidelberg, Germany; Madagascar Institute for Vaccine Research, University of Antananarivo, Antananarivo, Madagascar; International Vaccine Institute, Seoul, Republic of Korea

**Keywords:** antimicrobial usage, consumption, multi-country, data collection, AMU, AMC indicators

## Abstract

Excessive or inappropriate antimicrobial use contributes to antimicrobial resistance, emphasizing the need to monitor and document the types and quantities of antibiotics used. Thus, data on antimicrobial consumption (AMC) and antimicrobial usage (AMU) are key in informing and promoting judicious use. Our study, conducted during 2019–2023, as part of the CAPTURA project, aimed to understand the state of data availability and quality for AMC and AMU monitoring in Asia. In this article, we describe the challenges and opportunities faced and provide examples of AMU and AMC analysis. World Health Organization (WHO) and country-tailored methodologies and tools were applied to collect retrospective data from 2016 to 2019 in Bangladesh, Bhutan, Laos, Nepal, Pakistan, Papua New Guinea, Sri Lanka, and Timor-Leste. The primary indicator for national AMC was total level of consumption, expressed as total defined daily doses (DDD) per 1000 inhabitants per day for the year or period of data collected. For facility AMC and AMU, the primary indicator was total DDD per admissions per day for the year or period of data collected. Although many countries faced infrastructural challenges in data collection and storage, we managed to collect and analyze AMC data from 6 countries and AMU data from 5. The primary indicators, and additional findings, were visualized to facilitate dissemination and promote the development of action plans. Looking ahead, it is crucial that future initiatives empower each country to establish surveillance infrastructures tailored to their unique contexts, ensuring sustainable progress in the fight against antimicrobial resistance.

## Teaser

This study highlights the experience of data collection, curation, and analysis for the CAPTURA project in target countries. We also present the volume of antimicrobial consumption (AMC) and usage (AMU) data collected and show examples of analyses for key indicators.

Antimicrobial consumption (AMC) and usage (AMU) play a critical role in driving the development and spread of AMR. Therefore, accurate and comprehensive data on antimicrobial consumption and use provide valuable insights into the volume of antimicrobials dispensed, prescribing practices, trends, and determinants of use, enabling evidence-based interventions and strategies to promote rational and appropriate use [1–3].

Antimicrobial consumption (AMC) data include country- or region-wide statistics of total sales or imports of antimicrobials and is often obtained through a national regulatory authority, pharmaceutical wholesaler, or from health insurance reimbursement data [4, 5]. Antimicrobial usage (AMU) data, on the other hand, is typically obtained through pharmacies or hospitals as prescription records of antibiotics dispensed. Such data can also include demographic and clinical patient-level information [4, 6].

The most common approach for surveillance of AMC and AMU is set forth by the World Health Organization (WHO), which has initiated global programs to provide countries with common methodologies for collecting and reporting standardized data [3].

GLASS-AMC, a module of the Global Antimicrobial Resistance and Use Surveillance System (GLASS), provides a method to present and compare AMC data at international, national, and regional levels [3, 7]. The WHO Anatomical Therapeutic Chemical (ATC) classification system and defined daily doses (DDDs) methodology are typically used to comparably monitor consumption across different settings.

The WHO Methodology for Point Prevalence Survey on Antibiotic Use in Hospitals and the Global PPS serve as the most common methods to gather AMU data. Common reporting AMU statistics are number of antibiotic prescriptions per antibiotic subclass, indication or hospital ward, and proportions of AWaRe antibiotic use in inpatients using the Access, Watch, and Reserve (AWaRe) classification. This classification is a tool that supports monitoring of antibiotic consumption and stewardship efforts at local, national, and global levels [8].

Fleming Fund's “Capturing data on Antimicrobial Resistance Patterns and Trends in Use in Regions of Asia” (CAPTURA) project joins the effort of increasing the volume of historical and current data on resistance and use of antimicrobials in eight countries in Asia. CAPTURA used WHO and country-tailored methodologies and tools to collate, curate, and analyze retrospective AMC and AMU data between the years 2019 and 2023 in Bangladesh, Bhutan, Laos, Nepal, Pakistan, Papua New Guinea, Sri Lanka, and Timor-Leste. Here we describe the challenges and opportunities we faced to advance monitoring, collection, and utilization of AMC and AMU data in the Asian region to mitigate the growing threat of AMR.

## METHODS

CAPTURA employed various methods to investigate the availability, quality, and utilization of retrospective AMC and AMU data in each country. This involved initial preparation, facility selection and engagement, and data collation.

### Initial Preparation

Prior to data collection, comprehensive country engagements were conducted to identify potential sites and understand the current state of AMC and AMU initiatives. This involved a landscape analysis, that included scientific literature reviews, consultations with local stakeholders, and analysis of country-specific government documents and reports. Existing AMR surveillance networks were also assessed, and collaboration with coordinating committees, hospitals, and laboratories within the network was established. In-country teams were recruited and formed to serve as the focal personnel for activities in each country during the study period [9].

### Data Collection and Tools

The in-country CAPTURA team collaborated directly with data-holding facilities and data collection efforts were tailored for each country depending on data type, availability, and recording system variations (electronic or paper-based). All collected data were uploaded to the CAPTURA warehouse, a file-sharing site with customized privacy settings for each country to upload, store and transfer their files.

In countries with electronic recording systems, the data were directly shared in Microsoft Excel files by local drug authorities or hospital pharmacies. However, in countries with paper-based recording systems, a CAPTURA AMU template was developed (broadly) in line with existing WHO guidelines and The WHO Methodology for Point Prevalence Survey on Antibiotic Use. The template was used by the facilities to digitize prescription records and includes variables such as geographic information, patient demographics, and clinical information.

The AMU template was designed to aid data entry by limiting the use of free text. It included built-in validation checks, lists, and Excel macros that generated standardized drug utilization metrics. During the digitization process, in-country teams would share sample data with the data managers to confirm appropriate collection. The AMU template and completion guide for data collection can be found in Supplementary Files 1 and 2, respectively.

The collected data that contained patient information were encrypted and anonymized using a CAPTURA developed Data Formatting Tool (CAPTURA hashing tool [ivi-data-{PI}{PI}tools.azurewebsites.net]). The tool also ensured the data files contained only specified variables, with all other fields automatically removed.

**Figure 1.**
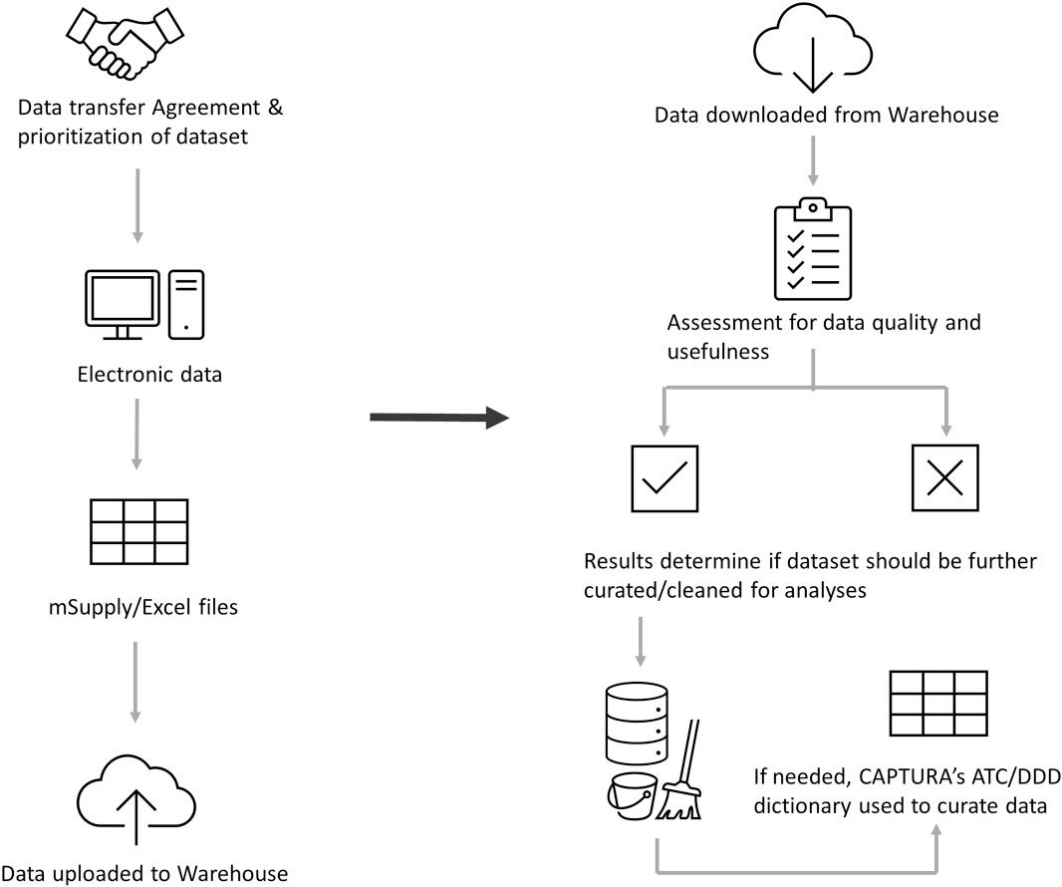
AMC data collection and curation process. Abbreviations: AMC, antimicrobial consumption; ATC, Anatomical Therapeutic Chemical classification system; CAPTURA, Capturing data on Antimicrobial Resistance Patterns and Trends in Use in Regions of Asia; DDD, defined daily doses.

**Figure 2.**
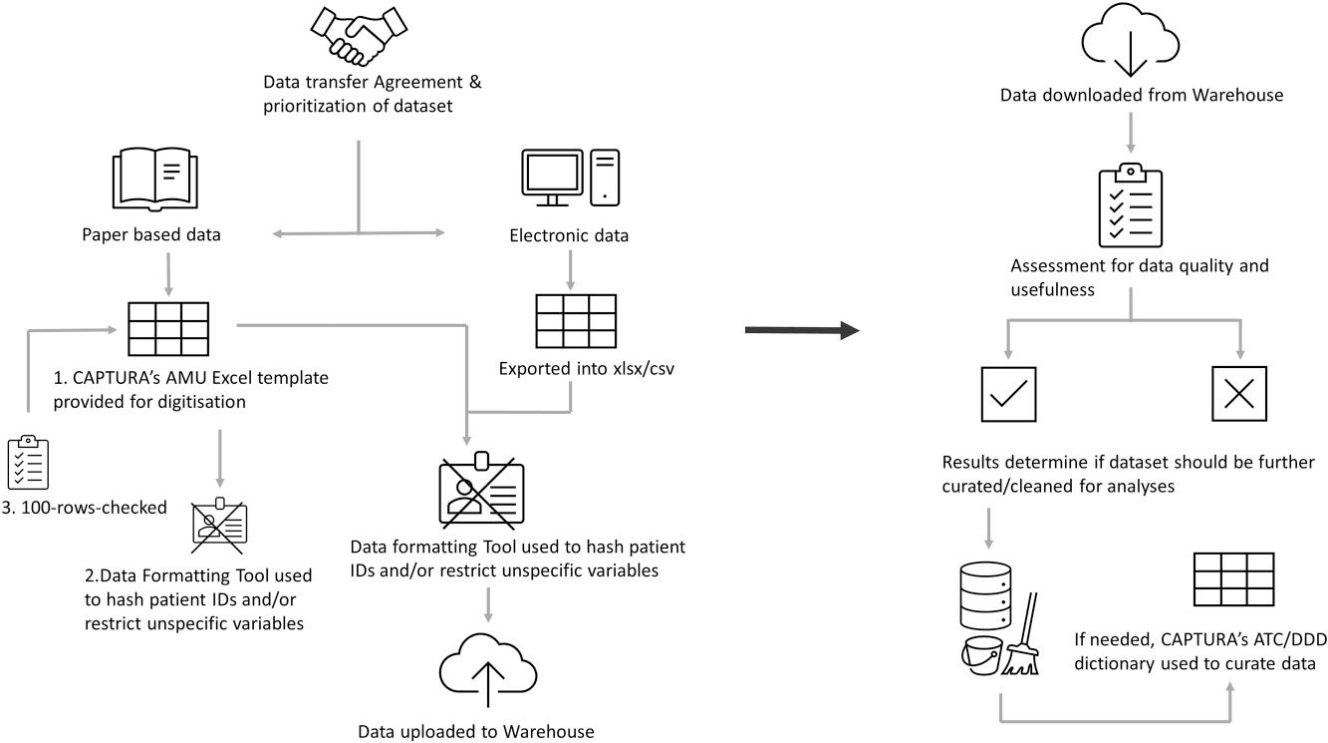
AMU data collection and curation process. Abbreviations: AMU, antimicrobial usage; ATC, anatomical therapeutic chemical classification system; CAPTURA, Capturing data on Antimicrobial Resistance Patterns and Trends in Use in Regions of Asia; DDD, defined daily doses.

Contextual information in the form of accompanying data documentation as “read me” files or hospital indicators were also collected for each dataset. Hospital indicator template can be found in Supplementary File 3. The data selection and collection process are illustrated as flowcharts in Figures 1 and 2 [10].

### Data Curation

Managing multiple facilities and handling numerous data sets from different countries, the team had to prioritize data curation efforts. The team's assessment of the datasets focused on the completeness, cleanliness, and structure of the data, with datasets that contained clinical information given highest priority. Curation efforts, to derive the AMC and AMU metrics, were tailored for each dataset.

Data cleaning, analysis and visualization were done using Microsoft Excel and R Software version 4.3.2 [11]. For some AMC data, visualization was performed through a CAPTURA developed open-source AMC visualization tool (AMC Dashboard [amc.qaapt.com]). The tool allows individual facilities or countries to build their own, individually tailored, and interactive AMC dashboard files in a user-friendly manner. CSV file format and specific variables are required to ensure proper visualization of the datasets.

### WHO Anatomical Therapeutic Chemical (ATC) Classification and Defined Daily Dose (DDD) Assignment

One important aspect of the data curation was extracting and linking the antimicrobials to the WHO's Anatomical Therapeutic Chemical (ATC) and Defined Daily Dose (DDD) System [7]. The ATC/DDD system is used to aggregate medicines data and serves as a tool for drug utilization monitoring and research. In this system, medicinal products are classified according to the main therapeutic use of the main active ingredient and are assigned only 1 ATC code per route of administration. Sometimes, a substance could be given more than 1 ATC code if available in more than 1 strength or route of administration with different therapeutic uses. An example relevant to CAPTURA antimicrobials is metronidazole, if prescribed orally or rectally it has an ATC code of P01AB01 (used for amoebiasis, trichomoniasis, and giardiasis) and parenterally as J01XD01 (used for treatment of anaerobic bacterial infections). Per ATC code and route of administration, only 1 DDD is assigned per drug and its route of administration. DDD values for the year 2019 were employed by CAPTURA, and this incorporated the significant revisions to the DDDs for antibiotics as recommended by the WHO Collaborating Center for Drug Statistics Methodology. A notable example of these revisions is the change in DDDs for amoxicillin and amoxicillin combined with β-lactamase inhibitors—key antibiotics in Antimicrobial Use (AMU) surveillance. Specifically, the DDDs were updated from 1 gram in 2018 to 1.5 grams in 2019 [7].

A dictionary containing the medicinal product name, the ATC codes, corresponding DDDs and mode of administration for all relevant CAPTURA categories was used to first extract the antimicrobials, then to attach the ATC codes and DDDs. The package “AMR” was also used as a dictionary for the ATC/DDD system [12].

For our study, the selected ATC categories included:

- ATC J01, antibacterial for systemic use
- ATC A07AA01-12, alimentary tract
- P01AB, metabolism and nitroimidazole derivatives against amoebiasis and other protozoal diseases

### WHO AwaRe Classification

The AWaRe classification is a framework developed based on the WHO Global Action Plan objectives and the WHO Model List of Essential Medicines that groups antibiotics into Access, Watch, and Reserve categories based on potential for development of resistance and treatment profile, to emphasize the importance of their appropriate use [13]. It is a useful tool for monitoring consumption, the effects of stewardship policies and defining targets that aim to optimize antibiotic use and curb antimicrobial resistance [8].

AMC data sets typically contained extensive information within a limited number of variables, necessitating the extraction of relevant antimicrobial information. Depending on the data received, strength, pack size, drug formulation, time period, and total number of drug distributions were cleaned and curated in preparation for analysis.

However, for AMU data, in addition to similar curation efforts to that of AMC data, patient information also had to be cleaned. For example, age (in years) was categorized into age groups: (eg, under 1 year of age, 1–5 years, 6–10 years, over 70 years), as well as gender, indications or diagnosis were cleaned or regrouped.

### Data Analysis

Aside from standardization, the ATC/DDD code system and AwaRe classifications also allowed for distribution and analysis of consumption and use.

### Antimicrobial Consumption

Our choice of primary AMC indicator was the total level of consumption, which was expressed as total DDD per year and DDD per 1000 inhabitants per day over the period of data collected.

Other Key indicators for AMC outputs at both national and facility levels were:

- Quantity of antibiotics as DDD per 1000 inhabitants per day (DID) for total consumption
- Quantity of antibiotics as DDD per 1000 inhabitants per day (DID) by pharmacological subgroup
- Quantity of antibiotics as DDD per 1000 inhabitants per day (DID) by AWaRe category
- Relative consumption of antibiotics as a percentage of total consumption by route of administration (oral vs parenteral)
- List of the most frequently used antibiotic substances.
  - Drug Utilization 75 (DU75) stratified by route of administration.
  - Top 10 by route of administration.

These indicators were visualized in the AMC dashboard to show trends in national, regional, and district levels, depending on the dataset uploaded (AMC Dashboard [amc.qaapt.com]). Using the AwaRe guidebook, the CAPTURA team used the WHO recommended target of 60% of antibiotic consumption to be in the Access group as a target statistic during analysis of national AMC data.

### Antimicrobial Usage

As a pilot study to understand the patterns of usage and appropriateness of antibiotic prescriptions, the WHO Methodology for Point Prevalence Survey on Antibiotic Use in Hospitals was used as a guide to collection, curation, and analysis of the AMU data [14]. During the AMU data analysis, the number of antibiotics prescribed in each facility was calculated, and trends were identified according to patient demographics, indication, the ward in which prescription was made, and the make-up of prescribed antibiotics according to the AWaRe classification and WHO ATC subgroups.

Key outputs of AMU analysis included:

- Overall number of antibiotics prescribed at facility level.
- Overall number of antibiotics prescribed in each facility, grouped by AWaRe and ATC pharmacological subgroup classifications.
- Number of antibiotics prescribed by medical wards.
  - Adult Medical Wards
  - Pneumology AMW
  - Adult Surgical Ward
  - Adult Intensive Care Unit
  - Pediatric Medical Ward
  - Neonatal Intensive Care Unit
- Number of antibiotics prescribed by ten most common diagnoses (in select facilities where this information was collected)
- Number of antibiotics prescribed by patient demographics (sex, age distributions)
- Number of antibiotics prescribed by indication.

Full rights to the data and analysis findings belong to the participating countries; therefore, in this article we have only highlighted major observations and have only included examples of AMC and AMU results using sample data, anonymized and aggregated data.

## RESULTS

### Antimicrobial Consumption

**Figure 3.**
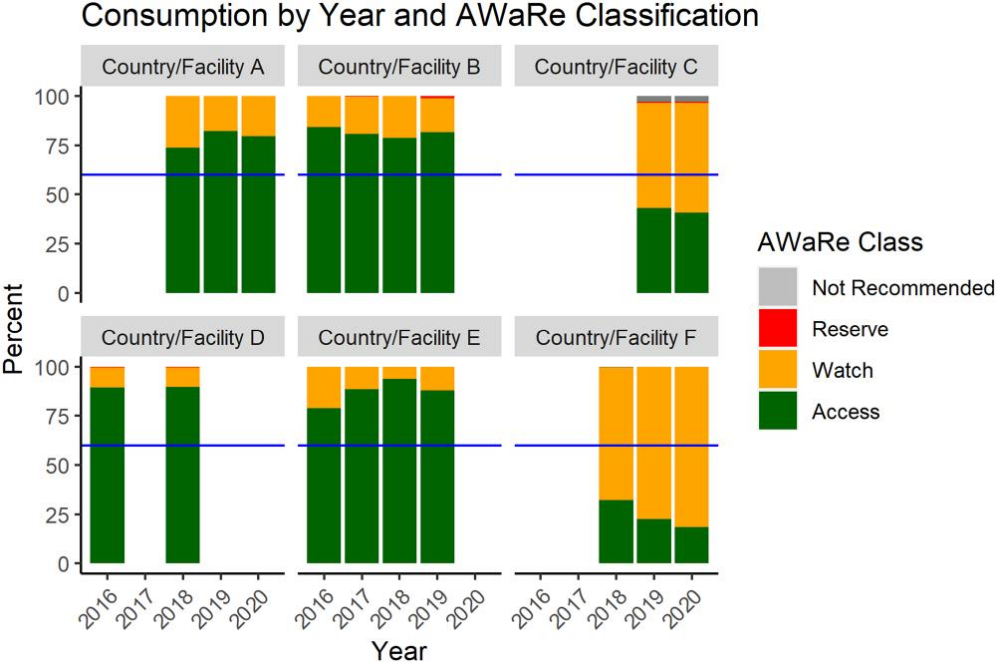
Proportion of AMC by AWaRe category, country/facility, and year. Abbreviations: AMC, antimicrobial consumption; AWaRe, Access, Watch, and Reserve.

The team was able to collect AMC data from 6 countries: Sri Lanka, Papua New Guinea, Pakistan, Timor Leste, Bhutan, and Laos. The data were mainly collected from public sources aside from Sri Lanka and Pakistan. The largest volume of AMC data (in terms of number of rows or observations) was collected in Sri Lanka, followed by Papua New Guinea and Pakistan. An overview of the data sources, coverage and type of data content found in the AMU and AMC data sets can be found in Table 1. In all countries, the team was able to conduct consumption analysis and used the AMC dashboard for visualization. Figure 3 displays national and/or facility AMC data of all the participating countries by year and AWaRe category. The columns in the graph represent percent proportion of the yearly national and/or facility consumption by AwaRe classification, with the horizontal line across representing the WHO country-level goal of using Access group antibiotics for at least 60% of all antibiotic usage. Four of the 6 countries or facilities were able to achieve this goal and even use more than the 60% recommendation. Three countries/facilities also used a small percentage of Reserve group antibiotics, and 1 country/facility had also used a very small percentage of antibiotics categorized as Not Recommended.

**Table 1.** Overview of the Data Sources, Coverage, and Type of Content in the Data Set

Data Type	Country	Geographic Area Covered	Source	Data^a^	Time Period	Observations
AMC	Bhutan	National	Public	Mixed Medical	2016–2017	380
					2018–2019	416
	Laos	National	Public	Antimicrobials	2018–2019	442
	Sri Lanka	Regional	Private	Antimicrobials	2018–2020	373 085
	Pakistan	National	Private	Antibiotics for systemic use (J01)	2019–2020	6628
	Papua New Guinea	National	Private	Antimicrobials	2016–2019	27 113
	Papua New Guinea	National	Public	Antimicrobials	2016–2019	2803
	Papua New Guinea	Regional	Public	Antimicrobials	2016–2019	1575
					2017–2019	225
	Timor Leste	Regional	Public	Antimicrobials	2016–2019	2963
	Timor Leste	National	Public	Antimicrobials	2016–2019	1332
AMU	Bangladesh	Regional	Private	Mixed Medical	2017–2020	1 664 941
					2016–2021	1 827 030
					2018–2021	49 034
					2017–2021	984 883
					2018–2020	2 901 652
	Bhutan	Regional	Public	Antimicrobials	2018–2019	3974
	Sri Lanka	Regional	Private	Antimicrobials	2018–2020	602 752
					2017–2019	155 819
	Nepal	Regional	Private	Antibiotics for systemic use (J01)	2017–2019	59 678
					2018–2019	2856
	Papua New Guinea	Regional	Public	Antibiotics for systemic use (J01)	2017–2019	20 437

Abbreviations: AMC, antimicrobial consumption; AMU, antimicrobial usage.

^a^The data column highlights the information found in the data sets.

A downloaded report, just like the ones shared with each CAPTURA country or facility can be found in Supplementary File 4. The results and visualizations for all AMC indicators presented in the report are from made-up data of a fictional country.

### Antimicrobial Usage

AMU data were collected from 5 of the 8 countries: Bangladesh, Sri Lanka, Nepal, Papua New Guinea, and Bhutan (see Table 1). The AMU data came mostly from private sources aside from Papua New Guinea and Bhutan. The largest volume of AMU data (in terms of number of rows or observations) were collected in Bangladesh, followed by Sri Lanka and Nepal. For some countries, not all AMU metrics were able to be generated considering the quality of the data, generally in such data sets the only patient related information was related only to demographics (age and gender). However, 2 countries were able to use the AMU template to digitize patient records found in logbooks. These data sets contained patient data expanding to patient treatment, diagnosis, and other clinical information. Some examples of similar AMU results as those presented to the countries can be found in Supplementary Files 5 and 6. All Supplementary Materials display numbers from a fictional hospital. Supplementary File 5 presents the percent proportion of treatment appropriateness by ward, where the patient's care has been reviewed by the hospital and deemed either appropriate, not appropriate or cannot be determined based on the available data. Supplementary File 6 displays antimicrobial prescriptions by ward and AWaRe categorization, where it is possible to determine the proportion of Access, Watch, or Reserve group antimicrobials used in each ward and inform on potential further investigation of prescription patterns in the wards.

## DISCUSSION AND CONCLUSION

The utilization of retrospective data for monitoring antimicrobial use can yield numerous benefits by offering a comprehensive view of past trends and patterns over time. This historical perspective provides a more precise understanding of antimicrobial prescribing practices and can aid in pinpointing areas that require improvement, whether in data collection or patient care [15]. By analyzing past trends and patterns, any shifts in the use of specific antibiotics or changes in prescribing guidelines can aid in monitoring the impact of interventions and policy changes [16].

Although retrospective data analysis is valuable for monitoring antimicrobial use, it also has limitations that need to be considered. Understanding these limitations is crucial for interpreting the findings accurately and making informed decisions.

The CAPTURA project faced several of these limitations during the data curation and analysis process.

Considering the reliance on existing records, some data sets were incomplete or contained missing information. Data entry errors, inconsistent recording practices, or variability in data formats across different healthcare settings hindered sufficient validation of the completeness and correctness of these existing data entries. Inaccurate or incomplete data can lead to biased results and hinder a comprehensive understanding of AMU patterns [17].

The data alone may not provide insights into the clinical context in which antibiotics were prescribed; therefore, understanding the rationale behind prescribing decisions and hospital practices is crucial for a comprehensive AMU assessment [18]. Considering the reliance on existing records, key contextual variables were either not recorded/available or not always collected in a coherent manner.

Because the data were often collected from specific healthcare settings or regions, with only few data sets containing a reliable national picture, the findings may not be representative of the entire population or different healthcare contexts, restricting the generalizability of the findings [19].

For AMU analysis, the team could not utilize the commonly used AMU protocols. The WHO methodology focuses on collecting a complete, expansive list of variables related to antibiotic prescription over a period of a day to a week, whereas for our analysis we collected retrospective data over 2–3 years. CAPTURA's approach to curating and analyzing AMU data were therefore loosely guided by the Global PPS methodology and hence was exploratory in nature and the findings primarily useful to inform the planning of prospective AMU surveillance, with the application of a rigorous methodology [20].

In addition to its work on the AMU data, the CAPTURA project also embarked on an analysis of Antimicrobial Consumption (AMC) using the Global AMC methodology. This approach allowed the project to gain a comprehensive understanding of antimicrobial usage patterns, complementing the AMU analysis. By applying the Global AMC methodology, the CAPTURA project was able to quantify the volume of antimicrobials used, providing valuable insights into the scale of antimicrobial consumption in the hospital setting. This dual approach, analyzing both AMU and AMC data for the same data sets, underscores CAPTURA's commitment to a thorough and multifaceted exploration of antimicrobial usage and consumption.

Moving forward, to enable both a high-quality AMU and AMC analysis CAPTURA recommends a standard data format with minimum variables, access to supporting documentation & tools, inclusion of clinical data including indication, diagnosis, and treatment. The variables not only add to the richness of the data but are necessary to ensure the quality of which this data is being recorded rather than focus on increasing the quantity of records [3, 6, 20].

Our study has demonstrated that large volumes of AMC and AMU data are available in the 8 countries engaged in Asia. With the collected data, we were able to show examples of analysis that can be conducted to inform the countries’ antibiotic use stewardship and treatment guidelines.

Each country exhibits varying levels of engagement with AMC and AMU surveillance initiatives, with many lacking appropriate infrastructures for storing information in easily analyzable formats. The top priority is implementing a well-managed record-keeping system and digitizing existing data using standardized codes. We recommend that future initiatives empower each country to spearhead efforts in building surveillance infrastructures tailored to their unique contexts. Concurrently, designing and launching national prospective AMC surveillance systems will be crucial for mitigating the further spread of AMR.

## Supplementary Data

Supplementary materials are available at *Clinical Infectious Diseases* online. Consisting of data provided by the authors to benefit the reader, the posted materials are not copyedited and are the sole responsibility of the authors, so questions or comments should be addressed to the corresponding author.

## Supplementary Material

ciad667_Supplementary_Data

## Notes

***Disclaimer.*** The views expressed here are those of the authors and not necessarily those of the UK Department of Health and Social Care or its management agent, Mott MacDonald; and, International Vaccine Institute.

***Authors Contributions.*** K. P. managed the data, curated, and analyzed it, and wrote the manuscript. K-y. K. C. was involved in data curation and analysis, conducted literature review, and provided critical revisions to the manuscript. E. E. provided guidance and supervision throughout the project and supported analysis interpretation. M. J. S. participated in data collection, performed data curation, and developed data curation and visualization tools. H. S. J. assisted with data collection and provided valuable feedback during manuscript preparation. N. P. assisted with data collection and provided guidance and supervision throughout the project. F. M. provided guidance and supervision throughout the project and revised the manuscript. M. H. provided guidance and supervision throughout the project and revised the manuscript for intellectual content.

***Acknowledgments.*** We extend our deepest gratitude to stakeholders from all countries participating in CAPTURA, CAPTURA in-country coordinators and teams as well as the data entry clerks whose dedication and hard work have been pivotal to the success of CAPTURA's project data collection. Special thanks are also extended to (in alphabetical order): 1) Directorate General of Drug Administration, Bangladesh 2) Department of Medical Services, Ministry of Health, Bhutan 3) Food and Drug Department, Laos 4) National Department of Health (NDoH), PNG 5) National Directorate of Pharmaceutical and Medicine and Servico Autonomo de Medicamentos e Equipamentos de Saude (SAMES) 6) Asiri Hospital Holdings PLC, Nawaloka Hospitals Research and Education Foundation and Lanka Hospitals Diagnostics (Private) Limited 7) Bir Hospital, Nepal Medical College, Vayodha Hospital and Sukraraj Tropical & Infectious Disease Hospital, Nepal.

***Supplement sponsorship.*** This article appears as part of the supplement “Capturing Data on Antimicrobial Resistance Patterns and Trends in Use in Regions of Asia (CAPTURA),” funded by the Department of Health and Social Care's Fleming Fund using UK aid.

***Financial support*.** This work was supported by the Fleming Fund regional grants (grant numbers FF10-135 and FF11-139). The Fleming Fund is a UK aid investment to support surveillance of antimicrobial resistance in low- and middle-income countries in Africa and Asia. The regional grants program is managed by Mott MacDonald Ltd, the Fleming Fund Grants Management Agent.
