# Supplementary material for: Collecting Multi-country Retrospective Antimicrobial Consumption and Use Data: Challenges and Experience
Source: Clin Infect Dis. 2023 Dec 20;77(Suppl 7):S528–35. doi: 10.1093/cid/ciad667 (PMC10732554; doi:10.1093/cid/ciad667)
Supplement: ciad667_Supplementary_Data [file ciad667_supplementary_data.zip › AMC Dashboard.html]

  AMC Dashboard You need to enable JavaScript to run this app.
