# Supplementary material for: Collecting Multi-country Retrospective Antimicrobial Consumption and Use Data: Challenges and Experience
Source: Clin Infect Dis. 2023 Dec 20;77(Suppl 7):S528–35. doi: 10.1093/cid/ciad667 (PMC10732554; doi:10.1093/cid/ciad667)
Supplement: ciad667_Supplementary_Data [file ciad667_supplementary_data.zip › Supplementary file 2_CAPTURA AMU Data Template Guide_ v1.5.pdf]

CAPTURA AMU DATA TEMPLATE

Before reading the rest of this instruction file & filling the template, please indicate the presence of the following variables from your data: A similar column recording the presence of the variables can be found in the second tab of the AMU Data Template excel file (Tab named AMU_Data_Dictionary).

!! Please make sure to record the presence of the variable through the checkbox**!!**

**If unclear on the meaning of the variable, please refer to the data dictionary of the second tab of the excel file and/or the dictionary found below on this document. **

Variable	Presence of the variable
COUNTRY	☐
ISO_CODE	☐
YEAR_of_DATA	☐
DISTRICT	☐
HOSPITAL	☐
DEPARTMENT	☐
WARD	☐
PATIENT_ID	☐
YEAR_of_BIRTH	☐
AGE_YEAR	☐
AGE_MONTH	☐
GENDER	☐
WEIGHT	☐
DRUG_GENERIC_NAME	☐
DRUG_CODE	☐
ATC_CODE	☐
DRUG_TRADE_NAME	☐
FORM	☐
ROUTE_ADMIN	☐
STRENGTH	☐
STRENGTH_UNIT	☐
DOSE	☐
DOSE_UNIT	☐
FREQ	☐
FREQ_UNIT	☐
START_DATE	☐
STOP_DATE	☐
TREAT_DURATION	☐
INDICATION	☐
INFECTION_SITE	☐
DIAGNOSES	☐

CAPTURA AMU DATA TEMPLATE

Introduction

This excel file is a template created following WHO guidelines on data collection for Antimicrobial Use.

In this excel file you will find 4 different tabs:

- AMU_Data_Template
- AMU_Data_Dictionary
- AMU_Annex I
- AMU_Drug_List_Annex II

**Please do not make any changes, deletions, or modifications of any kind to the template as you complete it. For ease of use, you may hide the variables ONLY. **

The **AMU_Data_Template** tab is the sheet where you will input and record the data you hold.

It contains the following 30 variables color coded to make completion easier for the user:

- Searchable drop- down list -> Yellow
- Automatically completed -> Blue
- Manual input -> Green

Variable	Input Method
COUNTRY	Searchable drop- down list
ISO_CODE	Automatically completed
YEAR_of_DATA	Searchable drop- down list
DISTRICT	Manual input
HOSPITAL	Manual input
DEPARTMENT	Searchable drop- down list
WARD	Searchable drop- down list
PATIENT_ID	Manual input
AGE_in_YEAR	Automatically completed
AGE_in_MONTH	Manual input
GENDER	Searchable drop- down list
WEIGHT	Manual input
DRUG_GENERIC_NAME	Searchable drop- down list
DRUG_CODE	Automatically completed
ATC_CODE	Automatically completed
DRUG_TRADE_NAME	Searchable drop- down list
FORM	Searchable drop- down list

ROUTE_ADMIN	Searchable drop- down list
STRENGTH	Manual input
STRENGTH_UNIT	Searchable drop- down list
DOSE	Manual input
DOSE_UNIT	Searchable drop- down list
FREQ	Manual input
FREQ_UNIT	Automatically completed
START_DATE	Manual input
STOP_DATE	Manual input
TREAT_DURATION	Automatically completed
INDICATION	Searchable drop- down list
INFECTION_SITE	Searchable drop- down list
DIAGNOSES	Manual input

FOR YOUR CONSIDERATION:

**Some columns will appear as #N/A, please do not be alarmed or deterred by this, it is just the way the formula appears when no information has been selected in its look-up cell.*

*Once you select an option from the DRUG_GENERIC_NAME &/OR COUNTRY variable, the corresponding information will appear in the neighboring cell. **

The **AMU_Data_Dictionary** tab has a description of all the variables in the template.

The **AMU_Annex I** tab contains various tables representing the variables with coded values and corresponding descriptions.

The **AMU_Drug_List_Annex II tab** contains a comprehensive list of the antibiotic names for systemic use, their unique three letter code identifier, and WHO ATC code.

STEP-BY-STEP INPUTTING METHOD:

ATTENTION:

Please do not make any changes, deletions, or modifications of any kind to the template as you complete it. For ease of use, you may hide the variables ONLY but remember to fill in “unknown/missing” if variable is not present.

If you have identified information that is unable to be recorded in the variables provided and would like to add any NEW variables, before sending it over to us or continuing to complete the template please PROVIDE us with the list of VARIABLE NAMES you would like to add ONLY as an extension to the already existing template.

**If you have any doubts, questions, or concerns please contact your IVI project coordinator. **

Instructions for coded and autocompleting variables

The header of the template has been frozen and shall remain static as you scroll down to input more information.

When clicking on the cell a facing down arrow will appear together with the whole list of values.

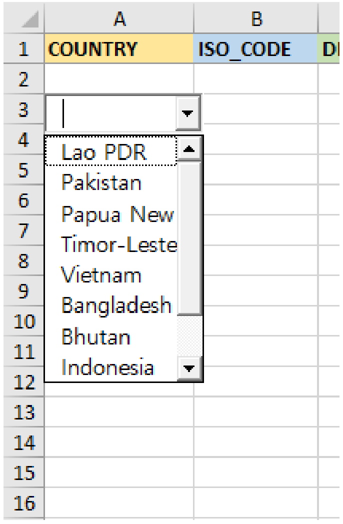

When clicking on the cell a second time, the cursor will appear, and you will be able to write the name of the drug you want to input. At this point, with each additional letter you type the list will get shorter as it matches the name.

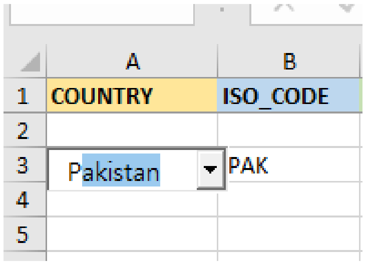

	A	B
1	COUNTRY	ISO_CODE
2		
3	Pakistan	PAK
4		
5		

For some of the variables, a corresponding one will automatically be completed based on the inputted value of the neighboring one.

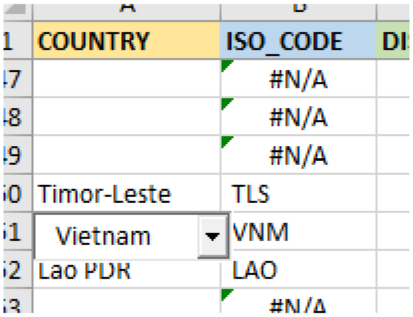

	A	B	
1	COUNTRY	ISO_CODE	DI
47		#N/A	
48		#N/A	
49		#N/A	
50	Timor-Leste	TLS	
51	Vietnam	VNM	
52	Lao PDR	LAO	
53		#N/A	

For the variable DRUG_GENERIC_NAME, two neighboring cells will autocomplete (DRUG_CODE & ATC_CODE).

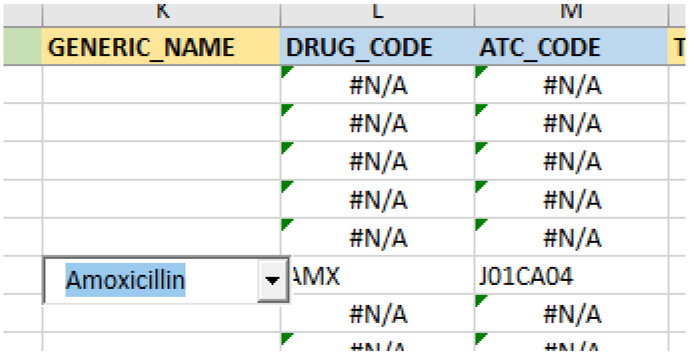

K	L	M	
GENERIC_NAME	DRUG_CODE	ATC_CODE	T
	#N/A	#N/A	
	#N/A	#N/A	
	#N/A	#N/A	
	#N/A	#N/A	
	#N/A	#N/A	
Amoxicillin	AMX	J01CA04	
	#N/A	#N/A	
	#N/A	#N/A	

When you first opened the file, you noticed that the autocompletion columns appeared as #N/A.This is no cause for alarm or error, hence please do not be deterred by this, it is just the way the formula appears when no information has been selected in its look-up cell. Once you select an option from the DRUG_GENERIC_NAME &/OR COUNTRY variable, the corresponding information will appear in the neighboring cell (as shown in the picture above).

Additional explanations to specific variables

In the second sheet of the Excel file is a variable dictionary (AMU_Data_Dictionary) that provides a more detailed description and examples of each variable. Below are some additional explanations for specific variables.

AGE & YEAR OF BIRTH

Please note, for patients *under the age of 2 years (24 months)*, please fill in <u>Age in Months</u> variable (0-24 months)

TREATMENT DURATION

<u>Treatment duration</u> is an autocompleted variable as a function of Start & Stop Date.

INDICATION & DIAGNOSIS

When recording the patient's condition, the <u>Indication</u> variable should be filled out FIRST. If selecting either "primary infection "or "Healthcare associated infection" from the drop-down list then proceed to fill in the <u>Infection Site</u> variable using its drop-down list in the next column. In case of another indication (all other options in "indication"), then please record the condition in the open text <u>Diagnoses</u> variable.

Tables summarizing the coded variables & their names are found in AMU_ANNEX I tab in the Excel Sheet and in the Annex of this document.

For a full list of all the antibiotics (trade name, generic name, ATC code) please refence the AMU_Drug_List_Annex II tab of the excel file.

**If unclear on what type of value should be inputted per variable, please refer to the data dictionary of the second tab of the excel file and/or the dictionary found below on this document*.*

*If you have any doubts, questions, or concerns please contact your IVI project coordinator. *

DATA DICTIONARY

Variable	Data type	Description	Remarks/Examples
COUNTRY	Coded Value (Text)	Country Name	
ISO_CODE	Coded Value (Text)	Three letter code uniquely identifying the reporting country	
YEAR_of_DATA	Coded Value (Date)	Drop-down of the 4 years of CAPTURA project period of interest	
DISTRICT	Text	District Name	
HOSPITAL	Text	Hospital Name	
DEPARTMENT	Coded Value (Text)	The department of the hospital (inpatient/outpatient)	
WARD	Text	Ward or Department Name	Surgical or pediatric
PATIENT_ID	Text	Unique Patient Identifier for the purpose of the study (no personal ID)	NO personal ID
YEAR_BIRTH	Number	Birth Year of the patient	
AGE_YEAR	Number	The age of the patient in years	Automatically completed as a function of the year of data recoded and the patient's year of birth
AGE_MONTH	Number	Age of pediatric patients	Up to 24 months
GENDER	Coded Value (Text)	Gender of patient (M or F)	
WEIGHT	Number	Weight of patient in kg (mainly for pediatric patients)	
DRUG_GENERIC_NAME	Coded Value (Text)	Name of drug (usually of active ingredient)	Azithromycin
DRUG_CODE	Coded Value (Text)	Three letter code uniquely identifying the drug	AZM
ATC_CODE	Alphanumeric	WHO ATC Code of drugs for systemic use (J01***)	J01FA10
DRUG_TRADE_NAME	Coded Value (Text)	Synonyms and trade names of antibiotics	Zithromax

FORM	Coded Value (Text)	The pharmaceutical form of the drug. (Formulation)	Tablet (TAB)
ROUTE_ADMIN	Coded Value (Text)	The route of administration of the drug.	Oral
STRENGTH	Number	The strength of the substance of each item as defined by PACKSIZE. For multi-ingredient products this field should contain the strength in which the DDD is expressed.	500
STRENGTH_UNIT	Coded Value (Measurement Unit)	The unit of the strength of the MPP	mg
DOSE	Number	The dose (amount that is taken) of drug the patient was prescribed or administered	
DOSE_UNIT	Coded Value (Text)	Unit of the dose (Tablets, mL, mg etc)	
FREQ	Number	The Frequency (how often) of the drug taken by the patient	
FREQ_UNIT	Coded Value (Text)	Time unit (day)	
START_DATE	Date	The date patient started to take the medicine	
STOP_DATE	Date	The date the patient stopped taking the prescription	
TREAT_DURATION	Number	The prescribed length of treatment in days	Automatic Completion as a function of the Stop and Start date of treatment
INDICATION	Coded Value (Text)	Options: 1. Primary infection; 2. HAI/HCAI (>48 h after admission); 3. Prophylaxis; 4. other/not applicable; 5. Unknown	Prophylaxis, given to prevent infection e.g., in association with surgery, catheter placement or other treatment
INFECTION_SITE	Coded Value (Text)	List of infections in major body systems (to be filled if indication is ‘primary infection’ or ‘HCAI’)	

ANNEX: Coded values and their meanings for your reference

Gender Code List	
CODE	***NAME***
M	male
F	female

Dosage Form/ Drug Delivery Code List	
CODE	***NAME***
TAB	tablet
CAP	capsule
SOL	solution
SYR	syrup
POW	powder
IV	intravenous injection
IM	intramuscular injection
SUS	suspension

Time Code List	
CODE	***NAME***
H	hour
D	day
W	week
M	month
PRN	as required
Y	year

Hospital Ward List
NAME
Medical
Emergency
Pediatrics
Intensive Care Unit
Surgical
Other
Unknown

Measurement Unit List	
CODE	***NAME***
mg	milligram
g	gram
mcg	microgram
IU	international unit
MU	millions of international unit
ml	milliliter
mmol	millimole
mg/ml	milligram/milliliter
UD	unit dose
PCS	piece

Administration Route List	
CODE	***NAME***
O	oral
P	parenteral
R	rectal
Inhal	inhalation
SL	sublingual/buccal/oromucosal
N	nasal
TD	transdermal
V	vaginal

ISO Country Code List	
NAME	***CODE***
Lao PDR	LAO
Pakistan	PAK
Papua New Guinea	PNG
Timor-Leste	TLS
Vietnam	VNM
Bangladesh	BGD
Bhutan	BTN
Indonesia	IDN
Nepal	NPL
Sri Lanka	LKA

Disease Category (Infection site)

Location	Examples
Bone and joint infection	Osteitis, pyogenic/purulent arthritis
Central Nervous System (CNS) infection	Meningitis
Ear infection	Otitis Media
Eye infection	Conjunctivitis
Gastro-intestinal infection	Salmonellosis, other enteritis
Genital (non-STI) infection	Cervicitis
Lower respiratory infection	Pneumonia, bronchitis
Upper respiratory infection	Tonsillitis
Skin and soft tissue infection	Skin infection, cellulitis
STI (sexually transmitted infection)	Gonorrhea
Urinary (non-STI) infection	Lower Urinary Tract infection, pyelonephritis
Systemic infection	Sepsis (bacteremia), endocarditis
Site of infection unknown	
Location	Examples
Bone and joint infection	Osteitis, pyogenic/purulent arthritis

Indication List

NAME	EXAMPLE
Primary infection	
HAI/HCAI (>48h after admission)	SSI (Surgical Site Infection), CAUTI (Catheter Associated Urinary Tract Infection), CLABSI (Central Line Associated Bloodstream Infection),VAP(ventilator associated pneumonia),HCAP (healthcare associated pneumonia)
Prophylaxis	Given to prevent infection e.g. in association with surgery, catheter placement or other treatment.
Other/ NA	
Unknown	
