# Supplementary figures and images for: Collecting Multi-country Retrospective Antimicrobial Consumption and Use Data: Challenges and Experience
Source: Clin Infect Dis. 2023 Dec 20;77(Suppl 7):S528–35. doi: 10.1093/cid/ciad667 (PMC10732554; doi:10.1093/cid/ciad667)

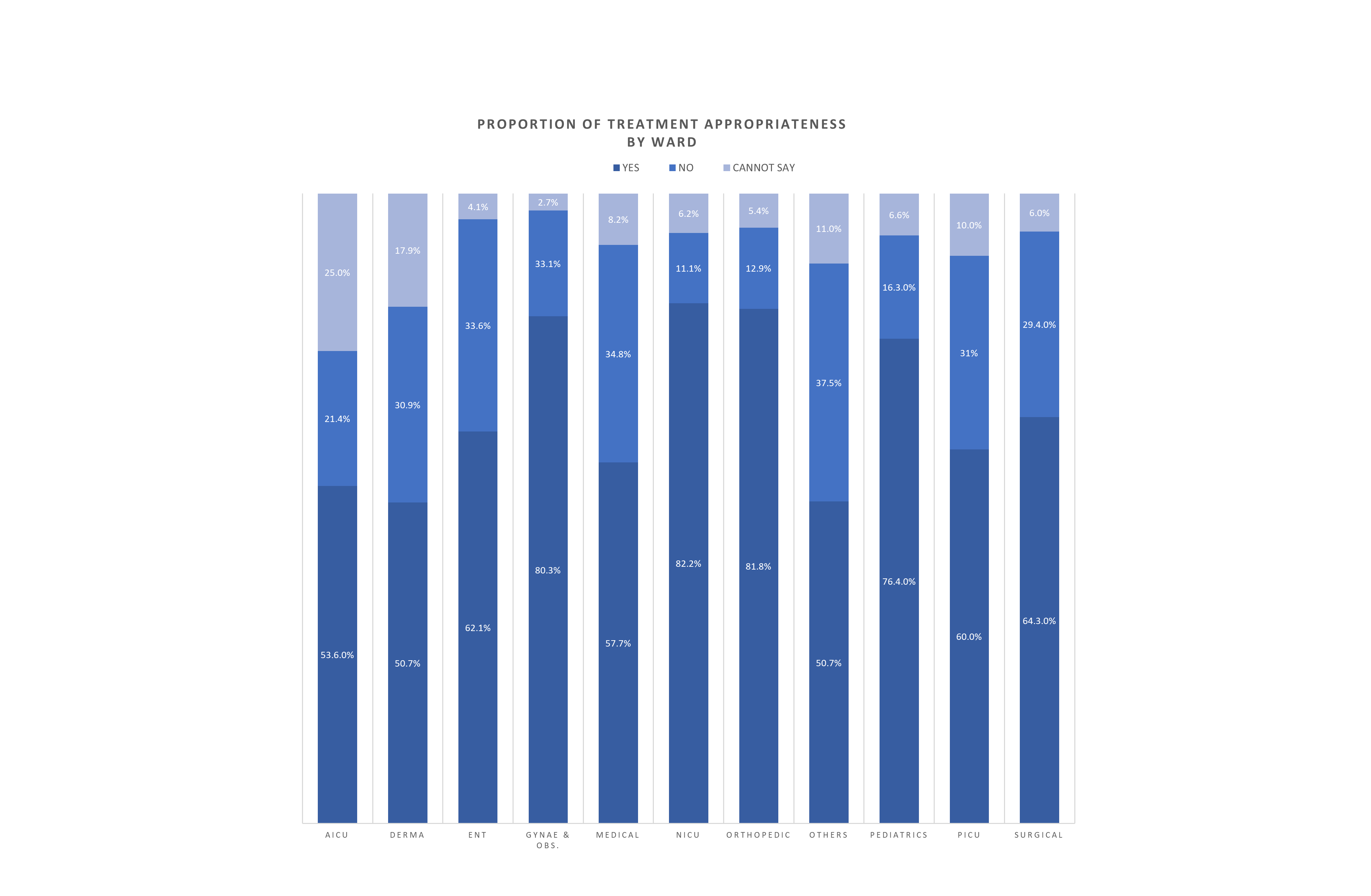

Supplement: ciad667_Supplementary_Data [file ciad667_supplementary_data.zip › 5. Prescription appropriateness by ward.tif]

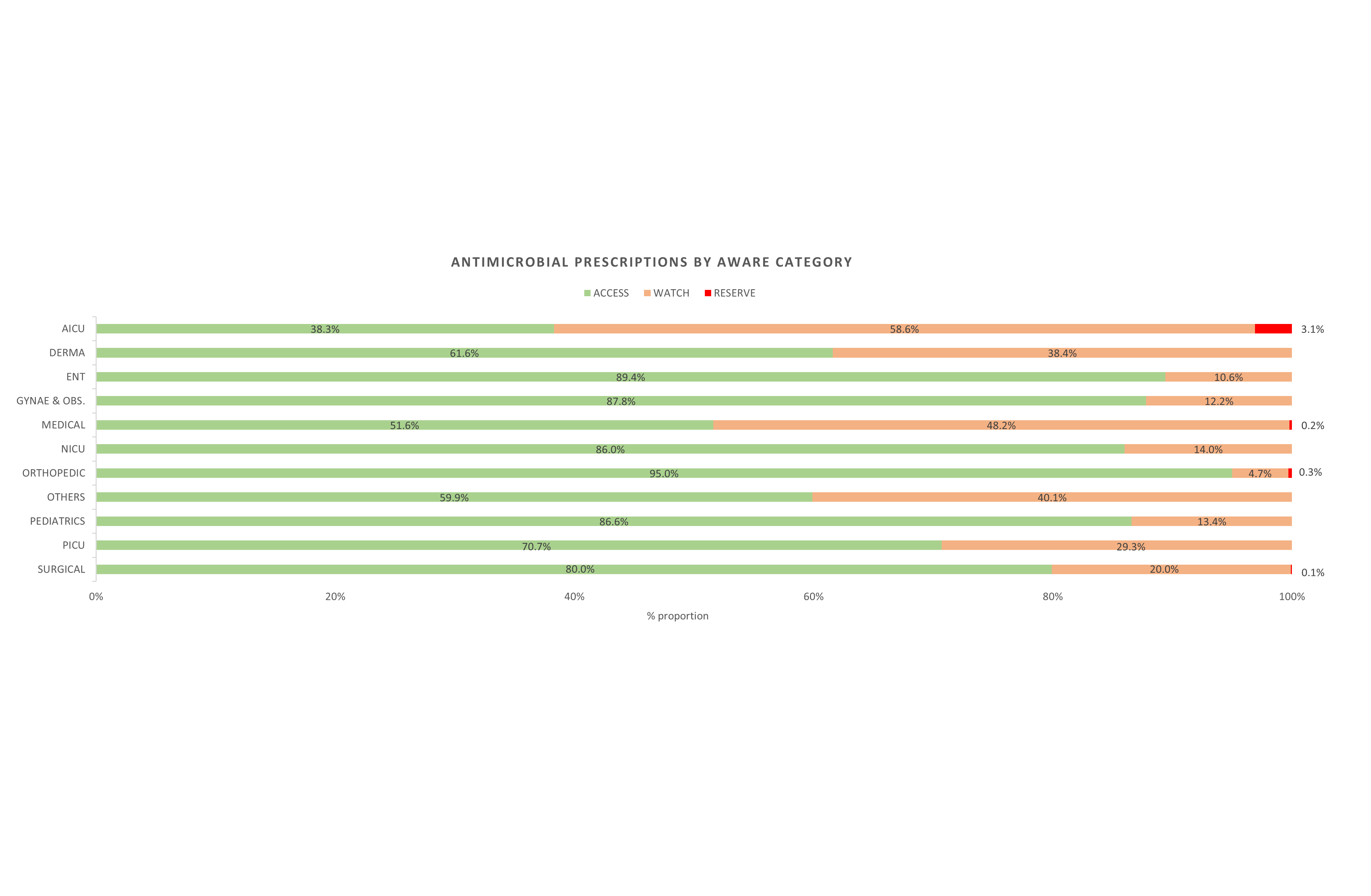

Supplement: ciad667_Supplementary_Data [file ciad667_supplementary_data.zip › 6. Prescriptions by AWaRe and ward.tif]
